# Combined Shear Wave Elastography and EU TIRADS in Differentiating Malignant and Benign Thyroid Nodules

**DOI:** 10.3390/cancers14225521

**Published:** 2022-11-10

**Authors:** Nonhlanhla Chambara, Xina Lo, Tom Chi Man Chow, Carol Man Sze Lai, Shirley Yuk Wah Liu, Michael Ying

**Affiliations:** 1 Department of Health Technology and Informatics, The Hong Kong Polytechnic University, Hung Hom, Kowloon, Hong Kong, China; 2 Department of Surgery, North District Hospital, Sheung Shui, New Territories, Hong Kong, China; 3 Division of Endocrine Surgery, Prince of Wales Hospital and Alice Ho Miu Ling Nethersole Hospital, Faculty of Medicine, The Chinese University of Hong Kong, Shatin, New Territories, Hong Kong, China

**Keywords:** thyroid nodule, ultrasound, shear wave elastography, Thyroid Imaging and Reporting Data System (TIRADS)

## Abstract

**Simple Summary:**

Grey scale ultrasound assessment is primarily used in the differential diagnosis of thyroid nodules. However, in recent years the assessment of tissue elasticity using shear wave elastography (SWE) has been suggested to have additional diagnostic value in thyroid cancer diagnosis. The aim of the present study was to evaluate the diagnostic efficiency of SWE in combination with grey scale ultrasound malignancy risk-stratification based on the European (EU) thyroid management guideline based on nodule size stratifications and indeterminate cytology status. We established that the combined approach had high diagnostic efficacy in differentiating malignant and benign nodules > 1 cm. However, in nodules with indeterminate cytology the approach highly discriminated benign nodules but resulted in low sensitivity which was indicative of an undesirable high false negative rate. The combination of SWE and the EU guideline is therefore most useful when evaluating non-micro-nodules based on size.

**Abstract:**

Although multimodal ultrasound approaches have been suggested to potentially improve the diagnosis of thyroid cancer; the diagnostic utility of the combination of SWE and malignancy-risk stratification systems remains vague due to the lack of standardized criteria. The purpose of the study was to assess the diagnostic value of the combination of grey scale ultrasound assessment using EU TIRADS and shear wave elastography. 121 patients (126 nodules–81 benign; 45 malignant) underwent grey scale ultrasound and SWE imaging of nodules between 0.5 cm and 5 cm prior to biopsy and/or surgery. Nodules were analyzed based on size stratifications: <1 cm (*n* = 43); 1–2 cm (*n* = 52) and >2 cm (*n* = 31) and equivocal cytology status (*n* = 52), and diagnostic performance assessments were conducted. The combination of EU TIRADS with SWE using the SD parameter; maintained a high sensitivity and significantly improved the specificity of sole EU TIRADS for nodules 1–2 cm (SEN: 72.2% vs. 88.9%, *p* > 0.05; SPEC: 76.5% vs. 55.9%, *p* < 0.01) and >2 cm (SEN: 71.4% vs. 85.7%, *p* > 0.05; SPEC: 95.8% vs. 62.5%, *p* < 0.01). For cytologically-equivocal nodules; the combination with the SWE minimum parameter resulted in a significant reduction in sensitivity with increased specificity (SEN: 60% vs. 80%; SPEC: 83.4% vs. 37.8%; all *p* < 0.05). SWE in combination with EU TIRADS is diagnostically efficient in discriminating nodules > 1 cm but is not ideal for discriminating cytologically-equivocal nodules.

## 1. Introduction

Grey scale ultrasound remains the first-line pre-operation diagnostic imaging method for thyroid cancers while fine needle aspiration cytology (FNAC) is regarded as the pre-surgery reference standard for the diagnosis of thyroid nodules. Ultrasound feature assessment has challenges of the overlap of some ultrasound features in benign and malignant nodules, as a result no sole feature is highly predictive of malignancy [1] On the other hand, although the sensitivity and specificity of FNAC can both range up to over 90%, it has challenges of non-diagnostic results in about 10% of the samples, and equivocal results in up to 30% of cases [2,3]. Different Thyroid Imaging Reporting and Data systems (TIRADS) have evolved to aid malignancy risk estimation and FNAC selection of thyroid nodules based on multiple grey scale ultrasound features and nodule size. Nonetheless, the diverse malignancy risk stratification criteria amongst different TIRADS result in varying diagnostic accuracy outcomes, thereby causing the lack of a universal standard for clinical use.

Elasticity imaging has been suggested as a useful complementary imaging modality that can improve the specificity and overall accuracy of grey scale ultrasound assessment in thyroid nodule diagnosis in addition to FNAC assessment [4,5].The evaluation of thyroid nodule stiffness as indicative of malignancy is purported to result in higher diagnostic accuracy when compared to different TIRADS [6,7]. Quantitative approaches such as shear wave elastography (SWE) that result in absolute tissue stiffness values rather than relative values or ratios are purported to be more objective and less user-dependent than strain elastography. Although SWE has gained popularity in recent years, its utility for thyroid cancer detection has been hampered by the lack of standardized diagnostic criteria. There is a lack of consensus regarding the best SWE measurement parameters, corresponding cut-off points and the SWE measurement techniques for the best diagnostic efficiency. Recent meta-analysis data showed different optimal cut-off ranges of between 27.7 to 85.2 kPa for the mean SWE index which corresponded with sensitivity and specificity ranges of 53% to 95% and 70% to 99%, respectively [8]. In other studies, the maximum SWE index was reported as having the best diagnostic efficiency while the minimum and standard deviation (SD) indices were seldom reported as achieving the best diagnostic performance [9,10,11,12].

Multi-modal ultrasound assessment has been proposed as a solution to improving the overall diagnostic efficacy in differentiating thyroid nodules, more so nodules with equivocal cytology [13]. However, the diversity of methodological approaches in different studies has resulted in conflicting opinions regarding the diagnostic value of multi-modal ultrasound imaging in thyroid nodule diagnosis. Moreover, limited studies have shown conflicting evidence regarding the benefit of combining SWE with grey scale ultrasound features or TIRADS in the diagnosis of thyroid cancer. Some studies reported an improved diagnostic performance with combined assessment, while others suggested that grey scale ultrasound feature assessment alone sufficed [14,15,16,17]. Although SWE has been suggested to have diagnostic value in aiding the diagnosis of thyroid nodules with equivocal cytology [18,19], the limited current studies exhibit similar challenges. Molecular tests are most accurate in diagnosing nodules with equivocal cytology; however, they are very expensive and not easily accessible [20].

Based on findings from our previous studies which demonstrated optimal diagnostic performance using EU TIRADS for thyroid cancer detection based on computer-aided and computer-assisted subjective assessment approaches [21,22], the present study evaluated the diagnostic efficiency of SWE in combination with EU TIRADS in thyroid nodule diagnosis based on overall nodule assessment, subcategory analyses by nodule size stratification and equivocal cytology status.

## 2. Materials and Methods

In this prospective study, cross-sectional cohorts of patients with thyroid nodules or thyroid cancer suspicion were purposively recruited from the Prince of Wales Hospital Department of Surgery and its affiliates using a consecutive case analysis approach and non-probability sampling. The Human Subjects Ethics Sub-Committee of The Hong Kong Polytechnic University granted the ethical approval (Registration Number: HSEARS20190123004) and the study was conducted between May 2019 and August 2021. The study adhered to the guidelines of the Declaration of Helsinki and patients provided informed consent before the data collection procedures.

### 2.1. Inclusion and Exclusion Criteria

All consenting adult patients (≥18 years old) with thyroid nodular disease or cancer suspicion who were booked for FNAC and/or subsequent surgical procedures, were included in the study. Nodules that were between 5 mm and 50 mm were included in the study. The lower limit for the nodule size is consistent with the size criteria for FNAC recommendation in addition to the clinical or sonographic risks when using most TIRADS. The upper limit was determined as the largest size that the transducer footprint can cover and can also be completely encompassed by the SWE acquisition colour box. In cases of the presence of multiple thyroid nodules, the nodule/s recommended for biopsy/surgery followed by the ones with the most suspicious sonographic features (microcalcifications, increased hypoechogenicity, irregular margins, tall-than-wide, etc.) were included in the study. Where there were no obvious suspicious features then the largest nodule was included in the study. Patients who were <18 years old, had history of a thyroidectomy, had a multinodular disease process without distinct isolated nodules and those that had no conclusive diagnosis based on cytology or histopathology results or both, were excluded from the study. Patients with only completely cystic nodules or nodules that were too large for the footprint of the transducer and could not be completely visualized in the image field of view were excluded from the study as they would affect the elastography output [4].

### 2.2. Ultrasound Imaging Procedures

Using one Aixplorer ultrasound machine (Supersonic Imagine, Aix-en-Provence, France) in conjunction with a 7–10 MHz linear transducer and SWE, a diagnostic radiographer with over 3 years of experience solely performed the thyroid ultrasound imaging, of all patients. To ensure consistency, this sole investigator standardized the ultrasound machine settings and maintained the same thyroid ultrasound scanning preset throughout the study.

The investigator followed standard ultrasound scanning protocols to conduct the thyroid scans. With each patient lying supine, minimal extension of the neck was applied, and the patient turned the face away from the side of interest. Coupling gel was applied to the exposed neck area of interest and the transducer was used to acquire 3 transverse and 3 longitudinal images of each target nodule in grey scale and SWE modes.

**Figure 1:**
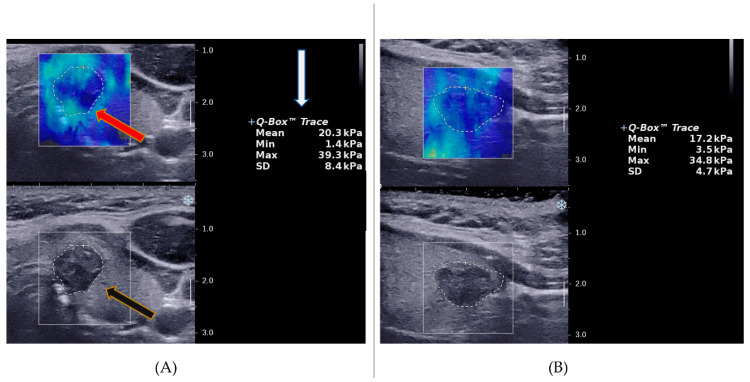
Image representation of the ROI selection for the quantification of stiffness in SWE. (**A**) An illustration of the Q-box placement and the nodule trace outline (red arrow), the output of the quantification of thyroid nodule elasticity in different SWE indices (white arrow) and the grey scale overlay image of the same nodule (black arrow) in the transverse plane. (**B**) The ROI trace representation and SWE quantification of the same nodule in the longitudinal plane. The scale on the right side of either image, represents the tissue depth from the point of contact of the transducer on the neck area, whereby 1.0 = 10 mm.

For the SWE mode, a generous layer of coupling gel was applied on the patient’s neck to minimize transducer compression on the neck and the static SWE images were acquired on arrested inspiration. The pre-set quantitative SWE measurement scale on the ultrasound machine ranged from 0 to 180 kPa. The SWE sampling box was adjusted to cover the whole thyroid nodule and the trace mode was used to manually outline the ROI. Images with intranodular cystic areas, calcified areas and areas void of SWE colour were avoided where possible. The inbuilt quantification tool, Q-box, automatically computed the minimum, maximum, mean and standard deviation (SD) elasticity indices in Young’s modulus (kilopascals- kPa) for each of the 3 images in the transverse and longitudinal planes (Figure 1). In the present study, the average values for each SWE index in kPa from the 3 images for each scan plane were compared against the final histopathology results to determine diagnostic accuracy.

### 2.3. Image Analysis Procedures

**Figure 2:**
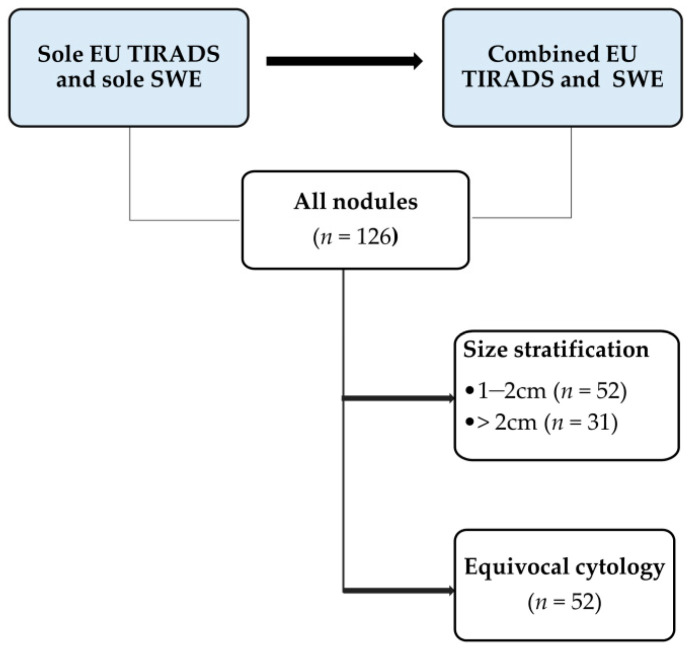
A stepwise illustration of the SWE analyses of the thyroid nodule images.

A stepwise approach was used to analyse the images based on each ultrasound modality used. The initial step involved analysing all thyroid nodules using grey scale ultrasound and SWE. Images were then distributed into relevant subcategories based on the nodule size stratification and equivocal cytology status. Figure 2 illustrates the analysis sequence and criteria. Nodules that had FNAC classifications 3 and 4 (atypia of undetermined significance or follicular lesion of undetermined significance-AUS/FLUS; and suspicion of follicular neoplasm or follicular neoplasm-SFN/FN, respectively) were considered equivocal [23,24]. The sole grey scale ultrasound assessment was based on EU TIRADS at a pre-determined cut-off point of high suspicion of malignancy (category 5).

### 2.4. Data Analysis and Statistical Analysis

Continuous data was classified as means +/− SD whereas categorical and/or nominal data was expressed as frequencies and percentages. The Chi-Square test was used to compare differences in nodule classification data. The Shapiro Wilk test was used to check the normality of the data. The Wilcoxon ranks test was used for the paired comparison of the SWE measurements between transverse and longitudinal planes while the Mann-Whitney U test was used to compare the SWE measurements between benign and malignant nodules.

The sensitivity (SEN), specificity (SPEC), positive predictive value (PPV), negative predictive value (NPV) and diagnostic odds ratio (DOR) were calculated with reference to final cytology or histopathology results. The receiver operating characteristic curves (ROC) were generated to obtain the area under roc curve (AUROC) and the optimal cut-off points for the SWE measurements were determined to be the maximum value of the sum of the specificity and the sensitivity of the AUROC. For the different subcategories of the thyroid nodules, the diagnostic performance measures were determined as follows:

(i) sole EU TIRADS and the average of each of the mean, maximum, minimum SD SWE indices
(ii) combination of EU TIRADS + each of the SWE indices at the determined cut-off values
(iii) sole EU TIRADS and each of the statistically significant SWE indices at the determined cut-off values for the different subgroups of the nodules
(iv) combination of EU TIRADS + SWE indices for the different subgroups of the nodules

For the combined assessment of TIRADS and SWE, a nodule was suspected of malignancy if the SWE value was ≥ the optimal cut-off point and had an EU TIRADS cut-off of ≥ category 4. The comparative analysis of sensitivity and specificity was conducted using McNemar and Cochran Q’s tests while comparison of the different AUROCs was done using the z-test. The tests were two-sided and *p* < 0.05 denoted statistical significance. The SPSS statistical software (version 26.0, SPSS Inc., Chicago, IL, USA) was used for the analyses.

## 3. Results

### 3.1. Demographics and Nodule Classification Data

**Figure 3:**
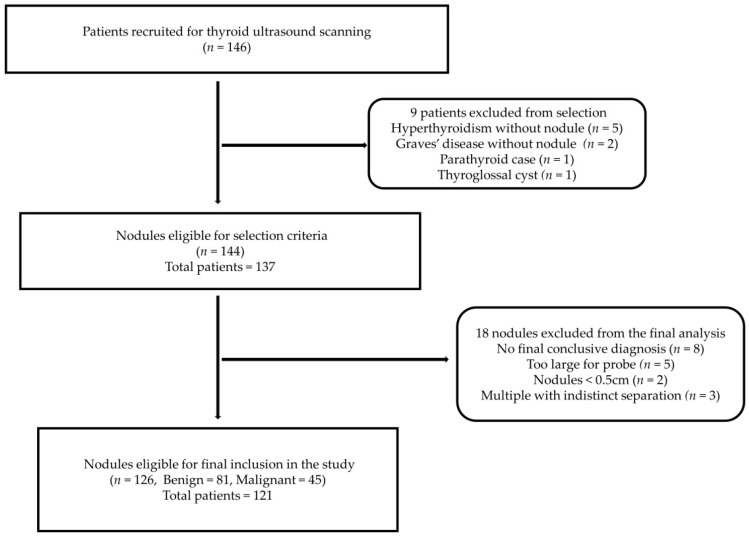
The image selection process of the ultrasound images of the thyroid nodules for the SWE study.

A total of 126 thyroid nodules (81 benign and 45 malignant) from 121 patients (100 females and 21 males) were included in the present study. Figure 3 shows the patient and thyroid nodule selection steps and exclusion reasons.

The mean age of the patients in this study was 53.8 ± 12.8 (range: 27 to 75) years. The mean age of male patients (62.1 ± 8.8, range: 44 to 73) was significantly higher than the mean age of female patients (52.1 ± 12, range: 27 to 75), *p* < 0.001. Table 1 shows the demographic data results of the patients. The mean nodule size was not significantly different between benign nodules (1.6 ± 0.8 cm, range: 0.5 to 3.6 cm) and malignant nodules (1.3 ± 0.8 cm, range: 0.5 to 3.7 cm), *p* > 0.05. The predominant nodule size category was 1 to 2 cm for benign nodules (65.4%) and <1 cm for malignant nodules (46.5%).

The classification of the nodules based on the cytology category revealed that 52 nodules (45.2%) had equivocal cytology of which 37 (71.2%) had benign histopathology results. The false-negative rate based on the FNAC was 4% (2/45). The common histopathology diagnosis of the malignant nodules was papillary thyroid carcinoma (PTC, *n* = 39), while the remaining nodules were classified as non-invasive follicular thyroid neoplasm with papillary like nuclear features (NIFTP, *n* = 2), follicular variant of PTC (FvPTC, *n* = 1), follicular thyroid carcinoma (FTC, *n* = 1), widely invasive FTC (*n* = 1) and minimally invasive FTC (*n* = 1).

### 3.2. Analysis of the Different SWE Indices in Thyroid Nodule Differentiation

#### 3.2.1. Comparison of SWE Index Medians Based on the Imaging Scan Plane

The medians of the different SWE indices were compared between transverse plane and longitudinal plane measurements for all nodules. The Wilcoxon signed rank test revealed that SWE measurements were significantly lower with the transverse plane for the mean and minimum indices than with the longitudinal plane (mean SWE index: median = 15.1 kPa vs. 17.9 kPa, z = −4.61, *p* < 0.001; minimum SWE index: median = 0.2 kPa vs. 1.4 kPa, z = −6.06, *p* < 0.001, respectively). The medians for the maximum and the SD SWE indices were not significantly different between the transverse and longitudinal scan measurements (maximum SWE index: median = 43.6 kPa vs. 42.3 kPa, z = −0.58, *p* = 0.56; SD SWE index: median = 7.2 kPa vs. 7.4 kPa, z = −0.27, *p* = 0.79, respectively).

#### 3.2.2. Comparison of SWE Index Medians between Malignant and Benign Nodules

The differences of the medians of the SWE measurement indices between benign and malignant nodules were evaluated with the Mann Whitney- U test based on different size stratifications and nodules with equivocal cytology status. Table 2 shows the *p* values of the statistical analyses of different SWE measurement indices and nodule subcategories. The transverse and longitudinal mean measurements, the longitudinal minimum, and the transverse SD measurement indices were statistically significant between benign and malignant nodules for all nodules and nodules of sizes between 1 to 2 cm (*p* < 0.05). The longitudinal minimum measurement index was statistically significant for the nodules with equivocal cytology (*p* < 0.05). The transverse SD measurement index was statistically different for nodules that were greater than 2 cm (*p* < 0.05). All SWE measurement indices did not differ significantly between malignant and benign for the nodules that were less than 1 cm (*p* > 0.05).

### 3.3. Diagnostic Performance Assessment of SWE Indices in Combination with EU TIRADS

The diagnostic performances of sole grey scale ultrasound assessment using EU TIRADS, sole SWE measurement indices, and combined EU TIRADS and SWE were evaluated for the different subcategories of nodules in the present study. Based on the subcategory of nodules with SWE indices that showed statistically significant differences between malignant and benign nodules, the optimal cut-off points of were determined and used in the diagnostic performance assessment (Table 3). Hence, the diagnostic performance of the SWE indices in nodules < 1 cm was not conducted in this present study.

#### 3.3.1. Overall Diagnostic Performance Assessment of SWE Indices for Evaluating All Nodules

Sole EU TIRADS achieved the highest sensitivity (84.4%), lowest specificity (51.9%) and the highest acceptable diagnostic efficacy (AUROC: 0.69) overall in diagnosing all nodules. Table 3 illustrates these results. The transverse mean (T_Mean_) SWE index resulted in diagnostic performance outcomes that were comparable to those of the longitudinal mean (L_Mean_) SWE index at the optimal cut-off, (SEN: 51.1% vs. 42.2%, and SPEC: 77.8% vs. 88.9%, *p* > 0.05). The diagnostic performance outcomes of the transverse SD (T_SD_) and the longitudinal minimum (L_Min_) indices (SEN: 51.1% and 53.3%, and SPEC: 76.5% and 76.5%, respectively) were comparable to those of both mean indices (*p* > 0.05). All the sole SWE indices had a significantly lower sensitivity but higher specificity than the sole EU TIRADS (*p* < 0.001). The combination of EU TIRADS and SWE maintained significantly lower sensitivities and higher specificities than EU TIRADS alone (*p* < 0.001).

#### 3.3.2. Diagnostic Performance Assessment of SWE Indices Based on Nodule Size Stratification

For nodules that were between 1 to 2 cm in size, sole EU TIRADS showed the highest sensitivity (88.9%) and the lowest specificity (55.9%). Out of all the SWE indices, the T_SD_ at an optimal cut-off of 8.7 kPa had the highest sensitivity (77.8%) and lowest specificity (64.7%) which were not significantly different from those of sole EU TIRADS (*p* > 0.05). The combination of EU TIRADS and SWE indices demonstrated that EU TIRADS + T_SD_ resulted in the highest sensitivity of 72.2% which was lower but not statistically significantly different from EU TIRADS alone (88.9%, *p* > 0.05) and a specificity of 76.5% which was significantly higher than that of EU TIRADS alone (55.9%, *p* < 0.01). The overall diagnostic efficacy of EU TIRADS + T_SD_ was the highest but still comparable to that of EU TIRADS alone (AUROC: 0.74 vs. 0.72, *p* > 0.05).

For the subcategory of nodules greater than 2 cm, the EU TIRADS maintained a high sensitivity but a lower specificity. The sensitivity was not significantly different from that of sole SWE T_SD_ at the optimal cut-off of 10.7 kPa and of the combined EU + T_SD_ approach (85.7% vs. 71.4% and 71.4%, *p* > 0.05) while the specificity was significantly different (62.5% vs. 83.3% and 95.8%, *p* < 0.01). The combined approach resulted in the highest diagnostic efficacy and comparably high predictive values (AUROC: 0.84, PPV: 83.3%, and NPV: 92%).

#### 3.3.3. Diagnostic Performance Assessment of SWE Indices in Discriminating Nodules with Equivocal Cytology

The diagnostic performance assessment of the 52 nodules with equivocal cytology revealed that the EU TIRADS alone resulted in significantly higher sensitivity but lower specificity than the sole SWE L_Min_ at the optimal cut-off of 6.1 kPa and the combined approach (SEN: 80% vs. 60% and 60%, *p* < 0.05; SPEC: 37.8% vs. 78.4% and 83.4%, *p* < 0.001). The overall diagnostic efficacy of the combined approach was significantly higher than that for EU TIRADS alone (AUROC: 0.72 vs. 0.58, *p* < 0.05).

## 4. Discussion

The present study evaluated the diagnostic value of SWE indices when combined with EU TIRADS based on the imaging scan plane, nodule size and in cytologically-equivocal thyroid nodules.

### 4.1. SWE Measurement Assessments Based on the Scan Planes

In the present study, only the mean SWE index demonstrated statistically significant differences between benign and malignant thyroid nodules using both scanning planes for all nodules and nodules between 1 to 2 cm in size. Although very few studies have evaluated SWE measurements concurrently in both scanning planes, our findings concur with two previous studies that suggested that a good concordance between transverse and longitudinal measurements can be achieved using the mean SWE index [15,25]. Nonetheless, their conclusions did not consider the influence of nodule size, which was done in the present study. The advantage of a SWE index whose measurements differ distinctly between benign and malignant thyroid nodules regardless of the scan plane used is the ability to still obtain accurate results using a scan plane best suited for a patient’s condition or nodule location. For example, imaging a nodule that is very proximal to the pulsating carotid artery in the transverse plane may result in stiffness measurement errors due to motion artifacts, however, these can be avoided by using the longitudinal plane [4,26]. Therefore, the routine clinical adoption of SWE requires standard cut-off measurement criteria for different scan planes which in turn may be influenced by the size of the nodule among other factors.

### 4.2. Diagnostic Performance of SWE Indices in Combination with EU TIRADS

#### 4.2.1. Analysis of All Nodules without Size Stratification

In the present study, the overall diagnostic performance evaluation of all nodules showed that no SWE index performed superiorly to EU TIRADS. This concurred with findings of Swan et al. [27] for which no SWE index outperformed the French TIRADS (an earlier version of the EU TIRADS) [19]. Some previous studies found the addition of SWE to grey scale ultrasound assessment with or without TIRADS to have no diagnostic value, while others found an improved diagnostic performance as evidenced by an increase in sensitivity and/or specificity [16,17,28,29,30,31,32]. The differences can be attributed to diverse study designs and SWE measurement methods. The present study used manual tracing for SWE measurement, while the previous studies used a fixed ROI and set it at either 2 or 3 mm placed over the stiffest portion of the nodule as determined from the qualitative elasticity colour scale. Although these conflicting findings suggest vague utility of the choice of the SWE measurement approach, total nodule tracing as used in the present study is suggested to be more reproducible with good intra- and inter-rater agreement [33]. However, the assessment of the stiffness of nodules of diverse size ranges cumulatively without size-stratification is a non-specific approach that likely contributed to no ideal diagnostic SWE index in overall assessment. This can be attributed to a wide range of potential cut-off points that were probably dependent on the range of the size of the nodules within the study.

#### 4.2.2. Analysis Based on Size Stratifications

The influence of the size of the nodule on combined diagnostic performance outcomes has not been extensively explored. Although some studies concluded that nodule size does not affect shear wave elasticity indices [34,35], similar to other recent studies, the present study found that nodule size may influence SWE indices [29,36]. The present study demonstrated that no SWE index resulted in a statistically significant difference in the SWE measurements between benign and malignant thyroid nodules < 1 cm. This concurred with some previous studies which concluded that malignant nodules < 1 cm may have low stiffness thereby resulting in poor discrimination from benign nodules using SWE [37,38,39]. Contrarily, Wang et al., [36] suggested that using the mean SWE index, the combination of SWE and grey scale ultrasound features resulted in improved specificity from that of sole grey scale ultrasound in nodules < 1 cm (SEN: 91.4% to 80.7%, SPEC: 66.7% to 77.8%). Meanwhile, a recent study suggested that the combination of the maximum SWE index at a cut-off of 28.2 kPa with any suspicious grey scale ultrasound feature had high sensitivity and specificity in discriminating sub-centimetre nodules [40]. The SWE ROI measurements were all different in the aforementioned studies, with one using 2 mm fixed ROI over the visually stiffest portion [41], another study using a circular Q-box to encompass the whole nodule [36] while the latter was based on perinodular or false rim stiffness [40].

For nodules of sizes between 1 to 2 cm and >2 cm, the T_SD_ SWE index at cut-offs of 8.7 kPa and 10.7 kPa, respectively, achieved the best diagnostic performance amongst the SWE parameters. The T_SD_ SWE index resulted in an improved specificity with minimal reduction in sensitivity when combined with EU TIRADS. Literature on diagnostic performance outcomes for SWE indices combined with TIRADS based on nodule size stratification is scant. However, in one study using the mean SWE index the combined assessment of SWE with TIRADS resulted in a higher sensitivity but reduced the specificity in nodules between 1 to 2 cm and nodules > 2 cm [36]. However, the cut-off points at which these diagnostic performance outcomes were not clearly stated. In a recent study, Li et al., [42] reported that the maximum SWE index in the longitudinal plane yielded high sensitivity and specificity at optimal cut-offs of 37.7 kPa for nodules > 1 cm and 55.1 kPa for nodules > 2 cm for (SEN: 96.4% and SPEC: 88.2%), respectively. However, their study evaluated sole SWE performance without the assessment of combined performance with TIRADS assessment. Contrarily, another study reported high diagnostic performance outcomes using the mean SWE index at a cut-off of 43.3 kPa for nodules between 1 to 3 cm (SEN: 82.8% and SPEC: 83.9%) and 42.7 kPa for nodules > 3 cm (SEN: 72.7% and SPEC: 91.4%) [41]. However, combined assessment with TIRADS was conducted only for all overall nodules but not for the size stratification groups in that study and the results showed no improvement in diagnostic performance.

The challenge of diverse SWE index parameters and optimal cut-off points is well-established in the literature. Different SWE measurement methods may explain the diversity. The present study established that the combination of EU TIRADS and the SD SWE index using the total nodular ROI tracing method may best discriminate nodules > 1 cm and >2 cm. As the SD SWE index may best represent heterogeneous fibrotic changes, it is likely more accurate when used using total nodule tracing for stiffness measurements. Since heterogeneous fibrotic changes are usually found in malignant nodules [43,44], the detection of these changes by the SD SWE index can help in differentiating benign and malignant nodules. However, fibrotic changes that result in elevated stiffness can also manifest in benign thyroid diseases such as different forms of thyroiditis and calcified multinodular goitres [43,45].Therefore, the SD SWE index in malignancy risk stratification may be most applicable when there is no suspicion or co-existence of these conditions in focal lesions.

Different thyroid management guidelines use the nodule size as one of the criteria to determine the treatment approach and recommend conservative treatment for indolent sub-centimetre nodules taking into account the patients’ preferences [20,46,47,48]. Hence, while the lack of size stratification yielded non-specific diagnostic utility findings in our present study, total nodular tracing is a more objective approach than subjective focal nodular ROI placement that demonstrates promise in improving SWE diagnosis when used with size-stratification. Therefore, we speculate that the complementary use of SWE with specific TIRADS will be beneficial when it is informed by the size of the nodule. Such an approach may limit the overdiagnosis of clinically insignificant sub-centimetre nodules for which active surveillance suffices rather than biopsy or surgery. Since the combination approach of EU TIRADS with SWE in the present study is based on methodological approaches that have ease of use and limited subjectivity, this could be advantageous for clinical implementation considerations.

#### 4.2.3. Analysis of Cytologically Equivocal Nodules

In the present study, the minimum SWE index in the longitudinal plane at the optimal cut-off of 6.1 kPa yielded high specificity (78.4%) with lower sensitivity (60%) with sole SWE analysis in cytologically equivocal thyroid nodules. Bardet et al. [49] and Samir et al. [50] reported a sensitivity that was comparably as high as the specificity (>80%) using a 65 kPa cut-off point for the maximum SWE index and a 22.3 kPa cut off point for the mean SWE index, respectively. However, Chen et al. [51] concluded that the SD SWE index at a cut-off of 3.3 kPa had the best diagnostic performance (SEN: 100% and SPEC: 49.8%) Nonetheless, that study included nodules with non-diagnostic cytology, which are not typically classified as equivocal. Furthermore, another study using a shear wave velocity maximum cut-off of 3.59 m/s achieved a higher sensitivity (83.9%) and a comparable but slightly lower specificity (79.2%) [52]. The study designs, sample sizes and SWE techniques and ROI measurements varied across the different studies thereby explaining the different study outcomes. Furthermore, while our study and the two other studies [50,52] only considered cytology categories 3 and 4 as equivocal, Bardet et al. [49] included the suspicion for malignancy (category 5). We excluded this category because of its inherent high risk of malignancy (60% to 75%) [24], which was 100% in our study upon final histopathology diagnosis.

The combination of EU TIRADS with SWE improved the overall diagnostic efficacy and specificity but lowered the sensitivity in the present study. Although due to sample size limitations sub-group analysis of the different equivocal cytology categories was not conducted in the present study, some previous studies reported EU TIRADS as diagnostically inefficient in the management of follicular neoplasms [53,54]. However, the diagnostic performance of different TIRADS in combination with SWE for differentiating cytologically-equivocal nodules has minimal exploration in the literature. Some previous studies suggested that the combination of strain elastography and/or SWE with grey scale ultrasound features had the potential for improving the diagnostic efficacy in cytologically equivocal nodules [55,56,57]. Contrarily, the combination of ACR TIRADS at category 5 with the maximum SWE index at a 41.2 kPa cut-off point had an outcome of raised sensitivity and reduced specificity in one study [58]. While the combination of ATA TIRADS at category 4 with the shear wave maximum velocity cut-off point of 3.35 m/s for cytology category 3 nodules, increased both sensitivity and specificity in another study [59]. The different study designs and SWE methodologies explain the differences in findings. The combination of EU TIRADS with SWE based on the selected cut-off points in the present study likely contributed to the reduction in the sensitivity. The findings may also be attributed to a low sample size which limited size-based analyses. Although the overall diagnostic efficiency improved significantly, the loss in sensitivity renders the diagnostic utility of this combined approach vague since the substantial reduction of sensitivity increases the false-negative rate thereby potentially delaying the treatment of cancer patients. Future larger prospective studies are warranted to validate and clarify the diagnostic value of this combined approach in cytologically-equivocal nodules.

## 5. Conclusions

The diagnostic performance of SWE in combination with EU TIRADS is influenced by the nodule size and has a good diagnostic value in nodules > 1 cm. Although the combination of EU TIRADS and minimum SWE index improved the specificity in equivocal nodules, the sensitivity was consistently and moderately lower than EU TIRADS alone thereby rendering the approach less ideal for routine clinical adoption.

## Figures and Tables

**Table 1:** Demographic data and the distribution of nodules into different classifications.

Characteristic	Overall Mean/Frequency	Mean/ Frequency by Diagnosis		*p*-Value
		B	M	
**Gender**				
Male	21	14 (66.7%)	7 (33.3%)	>0.05
Female	100	67 (67%)	33 (33%)	
**Mean Age**				
Overall	53.8 ± 12	53.8 ± 12.1	54.0 ± 12	>0.05
Male	62.1 ± 8.8			<0.001
Female	52.1 ± 12			
**Nodule size**				
Total nodules	126	81 (64.3%)	45 (35.7%)	<0.01
Overall mean (cm)	1.5 ± 0.8	1.6 ± 0.8	1.3 ± 0.8	0.62
<1 cm	43	23(53.5%)	20 (46.5%)	0.10
1–2 cm	52	34 (65.4%)	18 (34.6%)	
>2 cm	31	24 (29.6%)	7 (22.6%)	
**FNAC**				
Not done	11	11 (100%)	0 (0%)	<0.001
Non-diagnostic	6	5 (83.3%)	1 (16.7%	
Benign	30	28 (93.3%)	2 (6.7%)	
Equivocal	52	37 (71.2%)	15 (28.9%)	
Malignant/SOM	27	0 (0%)	27 (100%)	
**EU TIRADS**				
1	0	0 (0%)	0 (0%)	<0.001
2	27	24 (88.9%)	3 (11.1%)	
3	0	0 (0%)	0 (0%)	
4	22	18 (81.8%)	4 (18.2%)	
5	77	39 (50.7%)	38 (49.4%)	

B = benign; M = malignant; SOM = suspicion of malignancy, FNAC = fine needle aspiration cytology.

**Table 2:** Statistical significance assessment for the differences in SWE indices between benign and malignant thyroid nodules based on the scan planes.

Nodule Category	*p*-Values of SWE Indices in kPa							
	T_Mean_	L_Mean_	T_Min_	L_Min_	T_Max_	L_Max_	T_SD_	L_SD_
**All** T = 126 (B = 81, M = 45)	0.005 **	0.007 **	0.100	0.003 **	0.061	0.253	0.012 *	0.255
**Equivocal** T = 52 (B = 37, M = 15)	0.473	0.214	0.353	0.015 *	0.313	0.391	0.138	0.525
**<1 cm** T = 43 (B = 23, M = 20)	0.189	0.141	0.128	0.077	0.368	0.480	0.219	0.733
**1–2 cm** T = 52 (B = 34, M = 18)	0.017 *	0.010 *	0.865	0.195	0.034 *	0.102	0.009 **	0.108
**>2 cm** T = 31 (B = 24, M = 7)	0.104	0.661	0.216	0.835	0.061	0.417	0.029 *	0.085

SWE = shear wave elastography, T = total, B = benign, M = malignant, T_Mean_ = transverse Mean, LMean = longitudinal Mean, T_Min_ = transverse Minimum, L_Min_ = longitudinal Minimum, T_Max_ = transverse Maximum, LMax = longitudinal Maximum, T_SD_ = transverse Standard deviation, L_SD_ = longitudinal Standard deviation, EU = European, eqv = equivocal, *: *p* < 0.05, **: *p* < 0.01.

**Table 3:** Diagnostic performance assessment of sole and combined EU TIRADS and SWE indices based on all nodules, equivocal cytology, and size stratification.

Nodule Category	Diagnostic Test	Optimal Cut-Off	SEN (%)	SPEC (%)	PPV (%)	NPV (%)	AUROC
All	EU	5	84.4	51.9	49.4	85.7	0.69
	T_Mean_ (kPa)	19.3	51.1 ***	77.8 ***	56.1	74.1	0.65
	L_Mean_ (kPa)	28.2	42.2 ***	88.9 ***	67.9	73.5	0.65
	T_SD_ (kPa)	10.5	51.1 ***	76.5 ***	54.8	73.8	0.64
	L_Min_ (kPa)	4.7	53.3 ***	76.5 ***	55.8	74.7	0.66
	EU + T_Mean_		48.9 ***	82.7 ***	61.1	74.4	0.66
	EU + L_Mean_		40.0 ***	92.6 ***	75.0	73.5	0.66
	EU + T_SD_		51.1 ***	84.0 ***	63.9	75.6	0.68
	EU + L_Min_		51.1 ***	77.8 ***	56.1	74.1	0.64
Size 1–2 cm	EU	5	88.9	55.9	51.6	90.5	0.72
	T_Mean_ (kPa)	25.6	50.0 ***	94.1 ***	81.8	78.0	0.70
	T_Max_ (kPa)	50.2	61.1 **	73.5 **	55.0	78.1	0.68
	T_SD_ (kPa)	8.7	77.8	64.7	53.9	84.6	0.72
	L_Mean_ (kPa)	23.4	66.7 *	79.4 **	75.0	83.3	0.72
	EU + T_Mean_		44.4 ***	94.1 ***	80.0	76.2	0.69
	EU + T_Max_		55.6 ***	82.4 ***	62.5	77.8	0.69
	EU + T_SD_		72.2	76.5 **	61.9	83.9	0.74
	EU + L_Mean_		61.1 **	85.3 ***	68.8	80.6	0.73
Size > 2 cm	EU	5	85.7	62.5	40.0	93.8	0.73
	T_SD_ (kPa)	10.7	71.4	83.3 **	55.6	90.9	0.77
	EU + T_SD_		71.4	95.8 **	83.3	92.0	0.84
Equivocal	EU	5	80.0	37.8	34.3	82.4	0.58
	L_Min_ (kPa)	6.1	60.0 *	78.4 ***	52.9	82.9	0.64
	EU + L_Min_		60.0 *	83.4 ***	60.0	83.8	0.72 *

EU = European TIRADS, T_Mean_ = transverse Mean, L_Mean_ = longitudinal Mean, L_Min_ = longitudinal Minimum, T_Max_ = transverse Maximum, T_SD_ = transverse Standard deviation, kPa = kiloPascals, *: *p* < 0.05; **: *p* < 0.01; ***: *p* < 0.001 relative to EU TIRADS.

## Data Availability

The ultrasound and clinical data are publicly unavailable due to patient confidentiality reasons and privacy protection.
